# Thickness of hydrogel for nitrifying biomass entrapment determines the free ammonia susceptibility differently in batch and continuous modes

**DOI:** 10.1038/s41598-023-36507-4

**Published:** 2023-06-08

**Authors:** Minsu Song, Meng Yuan, Sanghyun Jeong, Hyokwan Bae

**Affiliations:** 1 Department of Civil and Environmental Engineering, Pusan National University, Busan, 46241 Republic of Korea; 2 Department of Urban and Environmental Engineering, Ulsan National Institute of Science and Technology (UNIST), 50 UNIST-gil, Eonyang-eup, Ulju-gun, Ulsan, 44919 Republic of Korea; 3 Graduate School of Carbon Neutrality, Ulsan National Institute of Science and Technology (UNIST), 50 UNIST-gil, Eonyang-eup, Ulju-gun, Ulsan, 44919 Republic of Korea

**Keywords:** Biological techniques, Environmental sciences

## Abstract

Hydrogels immobilizing nitrifying bacteria with different thicknesses of 0.55 and 1.13 cm (HG-0.55 and HG-1.13, respectively) were produced. It was recognized that the thickness of media is a crucial parameter that affects both the stability and efficiency of wastewater treatment. Batch mode experiments were conducted to quantify specific oxygen uptake rate (SOUR) values at various total ammonium nitrogen (TAN) concentrations and pH levels. In the batch test, HG-0.55 exhibited 2.4 times higher nitrifying activity than HG-1.13, with corresponding SOUR values of 0.00768 and 0.00317 mg-O_2_/L mL-PVA min, respectively. However, HG-0.55 was more susceptible to free ammonia (FA) toxicity than HG-1.13, resulting in a reduction of 80% and 50% in SOUR values for HG-0.55 and -1.13, respectively, upon increasing the FA concentration from 15.73 to 118.12 mg-FA/L. Continuous mode experiments were conducted to assess the partial nitritation (PN) efficiency in practical applications, where continuous wastewater inflow maintains low FA toxicity through high ammonia-oxidizing rates. With step-wise TAN concentration increases, HG-0.55 experienced a gentler increase in FA concentration compared to HG-1.13. At a nitrogen loading rate of 0.78–0.95 kg-N/m^3^ day, the FA increase rate for HG-0.55 was 0.0179 kg-FA/m^3^ day, while that of HG-1.13 was 0.0516 kg-FA/m^3^ day. In the batch mode, where wastewater is introduced all at once, the high accumulation of FA posed a disadvantage for the FA-susceptible HG-0.55, which made it unsuitable for application. However, in the continuous mode, the thinner HG-0.55, with its larger surface area and high ammonia oxidation activity, proved to be suitable and demonstrated its effectiveness. This study provides valuable insights and a framework for the utilization strategy of immobilized gels in addressing the toxic effects of FA in practical processes.

## Introduction

Nitrogen compounds are the most important nutrients that need to be removed from domestic and industrial wastewater^1^. The discharge of nitrogen compounds into water bodies leads to a decrease in dissolved oxygen, causing eutrophication and negatively impacting human health. Various methods have been developed for the removal of underwater ammonia. Some of these methods include air stripping^2,3^, chemical precipitation^4^, ion exchange^5^, filtration processes^6,7^, and biological nitrogen removal (BNR) processes^8^. Among them, the BNR process has gained widespread popularity due to its effectiveness in removing underwater ammonia through the activation of organisms. This approach offers several advantages, including enhanced efficiency, cost-effectiveness, and environmental friendliness, making it a preferred choice over other physicochemical processes^9,10^.

BNR process is primarily divided into the nitrification and denitrification stages. The nitrification stage, which is sensitive to factors such as pH, ammonia concentration, nitrite concentration, and temperature, is considered a rate-limiting step in the BNR process^11^. To overcome these limitations by improving the efficiency and stability of nitrification, various immobilization techniques have been developed to improve the efficiency and stability of nitrification^12^. The moving bed biofilm reactor (MBBR) uses whole cell immobilization to offer benefits such as low head loss, absence of filter channels, reduced need for backwashing, and a large surface area for colonization, resulting in high biological activity^13^. The properties of microbial carriers for application in the nitrogen removal process using MBBR are crucial, with key factors including large surface area, high mechanical strength, high porosity, and long durability^14^. In addition to the fundamental properties of the carrier, several studies have investigated the optimization of operating parameters for MBBR application, such as the packing ratio, dissolved oxygen (DO) concentration, and nitrogen loading^15,16^.

The application of whole cell entrapment as a technology for wastewater treatment has gained recognition as a reliable technology. Natural and synthetic polymers, including polysaccharides, polyacrylamide, and polyurethane, have been used for immobilizing living microbial cells. Previous studies have shown that poly (vinyl alcohol) (PVA), a low-cost and non-toxic polymer, is an effective option for entrapping nitrifying bacteria in wastewater treatment^17–19^. In these studies, the effectiveness of PVA hydrogel in removing total ammonium nitrogen (TAN) from wastewater has been demonstrated utilizing a hydraulic retention time of 6 h, resulting in up to 98% removal in wastewater with initial TAN concentrations ranging from 95 to 260 mg/L. In addition, PVA hydrogel has exhibited resistance to toxic chemicals such as high levels of chromium, leading to high removal rates of organic compounds, ammonia–nitrogen, and chromium^20^. However, microorganisms immobilized in chemically cross-linked hydrogels experience reduced activity due to the toxicity of the chemical agents. Additionally, for cryogels produced using freezing–thawing methods, ice crystal formation during the freezing process can damage microbial cell membranes. Therefore, prior to the actual application of immobilized nitrifying bacteria, enrichment is necessary to restore their activity.

The efficiency of nitrification per unit volume decreases as the hydrogel thickness increases due to the limited oxygen penetration, which is restricted to a magnitude of 1 mm in the PVA hydrogel^12^. In addition, the dense structure of PVA presents limitations in substrate and product transport in whole-cell entrapment^21^. When microbial biomass accumulates on the surface of hydrogel, the pores of the gel become clogged, restricting the infiltration of substrates into the inner regions^22^. In the event of hydrogel surface clogging, the hindered infiltration of substrates into the hydrogel's interior precludes any reactions from occurring. Therefore, it is crucial to increase the specific area per volume, and the simplest and most effective way to achieve this objective is the reduction of the thickness of the hydrogel^23^. To address this issue, reducing the size of the hydrogel can increase the external surface area, thereby enhancing the probability of contact between the substrate and the carrier per unit volume^24^. In addition, increasing the hydrogel's specific surface area and porosity can improve the permeability of the substrate into the gel^25^.

The partial nitritation-anaerobic ammonium oxidation (PN-AMX) process is an energy-efficient BNR technique that reduces costs associated with aeration and external carbon supplementation^26^. In this process, the ammonia-oxidizing bacteria (AOB) oxidize NH_4_^+^ to NO_2_^−^, and the AMX process requiring a ratio of 1:1.32 of NH_4_^+^ and NO_2_^−^^27^. The success of the PN-AMX system depends on achieving partial nitritation (PN) by activating the AOB and suppressing the nitrite-oxidizing bacteria (NOB), which is a key parameter in this process. Several operational conditions have been suggested, which encompass elevated temperature, increased pH level, the augmented concentration of free ammonia (FA), reduced aeration time, and lowered DO concentration^28^. FA results in have toxic effects on nitrifying microorganisms and its concentration is influenced by various factors such as TAN concentration, pH, and temperature^29^. In real wastewater treatment, these variables can change dynamically. Therefore, it's crucial to understand how nitrifying biomass performs under dynamic conditions to optimize treatment system design and operation. The toxicity of FA primarily disrupts the pH and ion balance by passing through the cell membrane^30,31^. The conversion of FA to ammonium leads to cell alkalization, which in turn triggers the pumping of protons by the cell to counteract the alkaline load^32,33^. This increased pumping activity results in higher energy demand. In addition, FA can inhibit bacterial enzymes and degrade extracellular polymeric substances (EPS). The breakdown of EPS makes the enzyme more susceptible to inactivation by FA^34^. The gel matrix used as a carrier has a wide gradient of DO concentration. Cells immobilized at depths of 10–230 μm from the surface of the hydrogel effectively maintain AOB activity while suppressing NOB activity^35^. Therefore, the degree to which different thicknesses of hydrogel influenced the activity of AOB and NOB through FA toxicity could vary.

Combining batch testing with methods such as response surface analysis (RSA) can be an effective way to analyze the characteristics of FA inhibition to nitrifying bacteria. The main benefit of batch experimentation resides in its ability to simultaneously evaluate multiple input variables, thus economizing on time and cost through a consolidated analysis of the resultant data^36,37^. This method increases the reliability of the results and allows for the identification of interactions between variables. However, this method has limitations in predicting the characteristics of microorganisms in continuous processes. In continuous mode, the system maintains low levels of FA to ensure optimal conditions for microorganisms to thrive. However, in batch mode, high concentrations of ammonia can be stressful for microorganisms, potentially leading to reduced activity levels and compromised results^38^. To treat high-concentration nitrogenous wastewater in a continuous mode, a strategy that gradually increases the nitrogen concentration from low to high has advantageous in terms of stability, as it maintains low levels of FA^39^.

The aim of this study is to investigate the inhibitory effect of PVA hydrogel thickness on nitrifying activity in batch and continuous modes. Because of the limited depth at which substrate, FA, and oxygen could penetrate into the hydrogel, variations in the thickness of the hydrogel may result in differences in the efficiency of PN due to differences in the activity levels of AOB and NOB. Thus, this study hypothesized that differences in hydrogel thickness could lead to significant differences in the efficiency of both nitrification and nitritation in practical applications. The nitrifying bacteria were enriched in PVA hydrogels of different thicknesses, and the specific oxygen uptake rate (SOUR) values were analyzed using TAN concentration and pH, both which determine FA concentration, as factors in RSA. The statistical mathematical approach of RSA can effectively analyze the complex variations of FA with TAN concentration and pH, and the consequent fluctuations in nitrifying performance by deriving a simulation model^40,41^. Based on the results of the RSA, simulations were conducted for TAN concentration and pH to revalidate FA inhibition. In addition, the inhibitory properties of AOB and NOB were examined by operating continuous stirred-tank reactors performing nitrification to evaluate the feasibility of the practical application of entrapped nitrifying bacteria to PN-AMX.

## Materials and methods

### Immobilization of nitrifying bacteria in PVA hydrogel

In this study, the inoculum of nitrifying biomass used was cultured under conditions of hydraulic retention time (HRT) of 24 h and an ammonia nitrogen concentration of 500 mg-N/L before being utilized. The ammonia removal efficiency was maintained at a level of 95% or higher with a nitrogen loading rate of 0.5 kg-N/m^3^ days. The nitrifying biomass collected from the inoculum reactor was immobilized in PVA hydrogel using the freezing–thawing method^42^. The PVA solution containing 20% PVA (with a degree of polymerization of 1500, JUNSEI, Japan) powder was autoclaved at 120 °C for 20 min to dissolve PVA completely. Nitrifying biomass of the same volume was taken and mixed with the PVA solution after the PVA solution was cooled down to 45 °C at room temperature. The final concentrations of PVA and volatile suspended solid (VSS) of nitrifying bacteria were 10% (w/v) and 955 mg/L, respectively. The mixture was poured into the rectangular plates at different depths to control the PVA hydrogel thickness. The PVA solution with nitrifying biomass was frozen at − 20 °C for 23 h and thawed at ambient temperature (20–25 °C) for 1 h. To enhance the mechanical strength of the PVA hydrogel, freezing and thawing cycles were repeated three times. Then, PVA hydrogel was placed in tap water to allow swelling for 24 h. Finally, the PVA hydrogel with different thickness was obtained. The average thicknesses of the obtained two types of PVA hydrogels were 0.55 ± 0.041 and 1.13 ± 0.030 cm (HG-0.55 and -1.13, respectively) (Fig. S1). The width and height of the square-shaped HG-0.55 and -1.13 were 1.25 cm.

### Enrichment process of nitrifying bacteria

40 ± 0.4 mL of fabricated PVA hydrogels with nitrifying biomass were packed in continuously stirred tank reactors. The reactors, with a working volume of 0.95 L, were aerated using air compressor at a flow rate of 1 L/min and maintained at a DO concentration of above 5 mg-O_2_/L. The HRT was fixed at 1.3 days. The temperature was fixed at 35 °C controlled by a water jacket. The synthetic ammonia wastewater was composed of 6 mg-P/L of KH_2_PO_4_, 12 mg-Mg/L of MgSO_4_·7H_2_O, 48 mg-Ca/L of CaCl_2_·2H_2_O, 1 mL/L of trace element solution I and 1 mL/L of trace element II. The trace element solution I was composed of 5 g/L EDTA and 5 g/L FeSO_4_·7H_2_O. The trace element solution II was composed of 5 g/L EDTA, 0.43 g/L ZnSO_4_·7H_2_O, 0.24 g/L CoCl_2_·6H_2_O, 0.99 g/L MnCl_2_·4 H_2_O, 0.25 g/L CuSO_4_·5H_2_O, 0.22 g/L Na_2_MoO_4_·2H_2_O, 0.19 g/L NiCl_2_·6H_2_O, 0.21 g/L Na_2_SeO_4_·10H_2_O and 0.014 g/L H_3_BO_3_. NH_4_^+^ and HCO_3_^−^ were added to a basal medium in the required amounts in the form of (NH_4_)_2_SO_4_ and NaHCO_3_, respectively. The required amounts of NH_4_^+^ and HCO_3_^−^ were added to a basal medium, using (NH_4_)_2_SO_4_ and NaHCO_3_, respectively. The ratio of HCO_3_^−^–C/NH_4_^+^–N is maintained at a ratio of 2:1 to achieve stable full nitrification^43^. The pH levels were not controlled.

### Analytical methods

The concentrations of NH_4_^+^–N, NO_2_^−^–N, and NO_3_^−^–N were measured using HUMAS test kit with a spectrophotometer (Think HS 3300, HUMAS, Republic of Korea). The samples were filtered through a 0.45 μm filter to exclude the effect of micro particles and chromaticity. The analysis of the samples was conducted promptly after sampling. The performance of the continuous modes with HG-0.55 and -1.13 was estimated by calculating the ammonia loading rate (ALR), ammonia removal rate (ARR), and nitrite removal rate (NRR), which are expressed in Eqs. (1)–(3), respectively^44,45^.

1 $${\text{ALR}}\left( {\frac{kg - N}{{m^{3} - d}}} \right) = \frac{{\left( {NH_{4}^{ + } - N} \right)_{Inf} \times 24}}{HRT \times 1000}$$

2 $${\text{ARR}}\left( {\frac{kg - N}{{m^{3} - d}}} \right) = \frac{{\left( {\left( {NH_{4}^{ + } - N} \right)_{Inf} - \left( {NH_{4}^{ + } - N} \right)_{Eff} } \right) \times 24}}{HRT \times 1000}$$

3 $${\text{NRR}}\left( {\frac{kg - N}{{m^{3} - d}}} \right) = \frac{{\left( {\left( {NO_{2}^{ - } - N} \right)_{Converted} - \left( {NO_{2}^{ - } - N} \right)_{Eff} } \right) \times 24}}{HRT \times 1000}$$

In the equations, “kg-N” indicates the mass of nitrogen in kilograms, “m^3^” represents cubic meters, “d” stands for day, "HRT" stands for hydraulic retention time (in days), “(NH_4_^+^–N)_Inf_” refers to the influent concentration of ammonia nitrogen, “(NH_4_^+^–N)_Eff_” represents the effluent concentration of ammonia nitrogen (in mg/L), “(NO_2_^−^–N)_Converted_” denotes the effluent concentration (in mg/L) of nitrite that has been converted from ammonia by AOB, and “(NO_2_^−^–N)_Eff_” indicates the effluent concentration of nitrite (in mg/L).

The DO and pH were measured by a DO meter (MultiLab 4010-1W interface, YSI, USA) and a pH meter (AB15 plus, Fisher Scientific, USA), respectively.

The FA concentrations were calculated by following equations^46^:

4 $${\text{NH}}_{{4}}^{ + } + {\text{OH}}^{ - } \; \leftrightarrow \; {\text{NH}}_{{3}} + {\text{H}}_{{2}} {\text{O}}$$

5 $$\left[ {{\text{NH}}_{{3}} - {\text{N}}} \right]_{{{\text{free}}}} = \, {{\left[ {{\text{TAN}}} \right]\left[ {{1}0^{{{\text{pH}}}} } \right]} \mathord{\left/ {\vphantom {{\left[ {{\text{TAN}}} \right]\left[ {{1}0^{{{\text{pH}}}} } \right]} {\left[ {{\text{K}}_{{\text{a}}} /{\text{K}}_{{\text{w}}} } \right] \, + \left. {{ 1}0^{{{\text{pH}}}} } \right]}}} \right. \kern-0pt} {\left[ {{\text{K}}_{{\text{a}}} /{\text{K}}_{{\text{w}}} } \right] \, + \left. {{ 1}0^{{{\text{pH}}}} } \right]}}$$

TAN = total ammonia nitrogen, ionization constant for NH_4_,

$$\left[ {{\text{K}}_{{\text{a}}} /{\text{K}}_{{\text{w}}} } \right]\; = \;{\text{exp}}\left[ {{6334}/\left( {{273 } + {\text{ T}}} \right)} \right],$$

K_a_ = ionization constant for ammonia (e.g., K_a_ at 20 °C = 10^−9.24^).

K_w_ = ionization constant for water (e.g., K_w_ at 20 °C = 0.69 × 10^−14^).

T = temperature in °C.

The FA concentration was theoretically calculated based on the TAN concentration, pH, and temperature for every condition of the SOUR experiments.

### Specific oxygen uptake rate of nitrifying bacteria

HG-0.55 and -1.13 were taken out from the enrichment reactors to evaluate the SOUR of nitrifying bacteria immobilized in PVA hydrogels. The hydrogels were washed with tap water to remove the sludge cake on their surface and then immersed in basal medium without NH_4_^+^ for one hour while being aerated at a 1 L/min intensity. SOUR analysis was conducted in batch mode, where hydrogels were added into glass bottles (316 mL) at a packing ratio of 1.28 ± 0.05% (v/v). The pH of medium was adjusted with 1 N HCl and 1 N NaOH for the designated experimental conditions. The DO concentration was recorded every 15 s for 10 min and these experiments were performed at 20 ± 0.4 °C. The slope of the decrease in the DO concentration was calculated by the linear regression of the least square method. The R^2^ values were sufficiently high (> 0.987) for all experimental conditions. Finally, the oxygen respiration rate was divided by the unit volume of hydrogels to calculate the SOUR. The SOUR experiment was duplicated for the feasibility test.

### Statistical analysis of the specific nitrifying activity

RSA was employed to assess the impact of critical factors (TAN and pH) on the inhibition of nitrifying activity. Various SOUR values, measured under different TAN concentrations and pH conditions, were applied as response values for RSA. The evaluation was conducted using the first-order, second-order, and partial cubic models within the ECHIP Software version 6 (ECHIP Inc., Delaware, USA). RSA is a tool for multivariate regression to develop a mathematical equation and find the optimal conditions of independent variables for the maximum or minimum response of dependent variables. RSA is an iterative statistical technique to approximate multivariate responses and is represented by the Eq. (6), as below^47,48^.

6 $$\eta \, = C_{0} + \mathop \sum \limits_{i = 1}^{n} \alpha_{{\text{i}}} x_{{\text{i}}} + \mathop \sum \limits_{i = 1}^{n} \alpha_{ii} x_{{\text{i}}}^{2} + \mathop \sum \limits_{i = 1}^{n} \mathop \sum \limits_{j > 1}^{n} \alpha_{ij} x_{{\text{i}}} x_{j} ,\;\;{\text{i}} < {\text{j}}$$

Here, η is the measured SOUR value of nitrifying activity [(mg-DO/L min) per mL-PVA], $x_{{\text{i}}}$ is the coded value of the *i*th test variable (1 = the NH_4_^+^–N concentration of medium (mg/L); 2 = pH), *C*_*0*_ is the regression constant, and $\alpha_{{\text{i}}}$ shows the regression coefficient of the independent variable i. The least squares method was used to estimate the parameters in the appropriate polynomials (Eq. 6). The experimental conditions for RSA were designed based on the central composite cube (CCC) design^49^.

For the two independent variables, there are 2^2(number of factors)^ corner points in addition to the central experimental condition. All these conditions are required for the first order model; then four axial points were added for the extended order of the regression. For the multivariate regression of RSA, measurements were conducted once under normal conditions and five times under central conditions (refer to Table 2). This type of design can minimize the number of trials required to obtain statistically significant results. NH_4_^+^-N concentration and pH ranges were designated from 29.3 to 170.7 and 6.6 to 9.4, respectively. Ultimately, the most suitable model for RSA was determined, and the corresponding regression equation was derived. Using this equation, the inhibition of FA toxicity for HG-0.55 and -1.13 was evaluated under various TAN concentrations and pH conditions.

### Experimental set-up of partial nitritation bioreactors

Two identical PN bioreactors were operated in continuous mode to compare with HG-0.55 and -1.13 for PN activity. The initial biomass concentration used for immobilization was 14,147 ± 92 mg-VSS/L for nitrifying bacteria. The HG-0.55 and -1.13 were inoculated in each bioreactor at a 10% (v/v) packing ratio respectively. The final biomass concentration in the bioreactors was 682 ± 2 mg-VSS/L. The operation temperature was controlled using the electric heat line and temperature controller. The airflow rate was maintained at l L/min using the air pump and a flow meter (LP-10A, Jungsu, Republic of Korea) to supply a sufficient amount of oxygen. The synthetic wastewater and mineral components were identical those used in the enrichment step. The influent was supplied by peristaltic pumps (NEXT 100M, NEXT PUMP, Republic of Korea), and the HRT was set to 1 day.

To initiate the AOB activation and suppress the NOB activation, the bioreactors were controlled systematically during phases I–IV (Table 1). To avoid the inhibition of nitrifying bacteria activity caused by the accumulation of FA, the influent TAN concentration was gradually increased. The concentration of NH_4_^+^–N was increased gradually from 100 to 1000 mg-N/L with 100 mg-N/L increments after more than 50% of the influent NH_4_^+^–N concentration had been oxidized. Because two times of bicarbonate is required for full oxidation of NH_4_^+^–N, HCO_3_^−^ was added as two moles of HCO_3_^−^ per one mole of NH_4_^+^ (Phase I in Table 1). In Phase II, HCO_3_^−^ was added 1–1.2 times based on moles of NH_4_^+^ to control AOB activity for adjusting NH_4_^+^–N conversion efficiency at 50%. To suppress the NOB activity, the temperature of the bioreactors was controlled at 35 °C, which resulted in a higher concentration of FA to suppress the NOB activity (Phase III in Table 1). In the final Phase IV, a high concentration of NH_4_^+^–N was added up to 1000 mg/L for evaluating whether PN activity could be maintained in an extreme environment with high-strength NH_4_^+^–N.

**Table 1:** Operational conditions of the PN bioreactor of HG-0.55 and -1.13 at each phase.

Phase	Operational strategy	Elapsed time (day)	Inf. NH_4_^+^–N (mg-N/L)	Ratio of HCO_3_^−^–C/NH_4_^+^–N	Temperature (°C)
I	Nitrification initiation period	1–26	100–300	2	Room temperature (20–25)
II	AOB activity control by adjusting alkalinity	27–57	300–500	1	Room temperature (20–25)
III	NOB inhibition by increasing temperature	58–87	500	1–1.2	35
IV	Exposure to high-strength ammonia	88–136	500–1,000	1–1.2	35

## Results and discussion

### Enrichment of nitrifying activity

The nitrifying activity of the two types of PVA hydrogels were monitored for 40 days during the enrichment (Fig. S2). High ammonia-oxidizing efficiency of over 96% was achieved, which was assisted by the entrapped nitrifying bacteria in PVA hydrogels. The different size of PVA hydrogels resulted in different NO_2_^–^–N oxidation efficiency while NH_4_^+^–N oxidation was stable for both thicknesses. At the end of the enrichment, complete nitrite oxidation was shown for the HG-0.55, but the HG-1.13 showed an incomplete nitrite oxidation (18.5%). In a previous study, a thickness of the attached growth system was identified as the key control parameter for suppressing NOB activity^50,51^. As NOB is more sensitive to DO concentration than AOB, the diffusivity of DO in the biofilm was found to have a significant impact on NOB activity. Likewise, the lower nitrite oxidation of HG-1.13 is attributed to the larger thickness. It was considered that NOB could not utilize oxygen substrate due to the limited specific surface area of HG-1.13 (3.66 cm^2^/cm^3^) which is smaller than that of the HG-0.55 (4.97 cm^2^/cm^3^).

### Effect of total ammonia nitrogen concentration and pH

Preliminary feasibility tests were implemented to determine the central experimental conditions of TAN concentration and pH. In order to conduct tests under conditions where the activity of nitrifying bacteria is sufficiently high, HG-0.55 and -1.13 were enriched prior to performing the SOUR test. The TAN concentration and pH were adjusted in the range of 50 to 500 mg-N/L and 5–9, respectively, based on the hypothesized optimal condition (e.g., TAN concentration of 100 mg-N/L and pH of 7).

**Figure 1:**
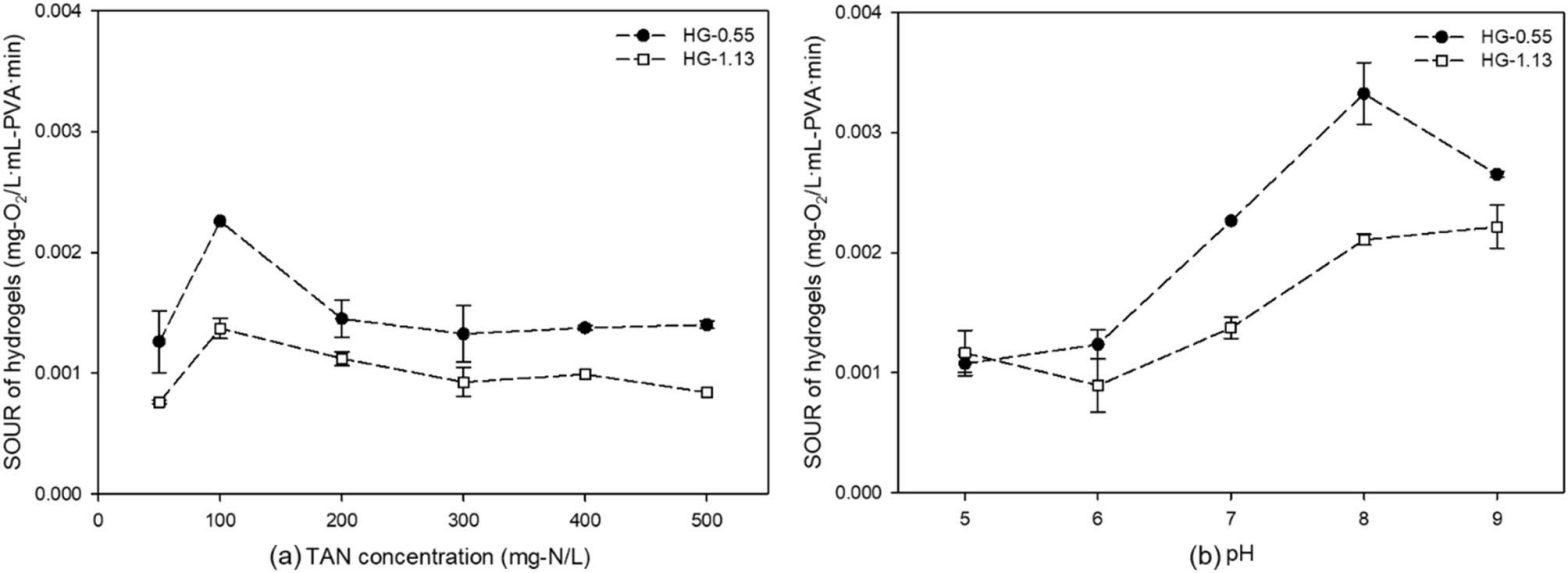
The SOUR of nitrifying bacteria immobilized in HG-0.55 and -1.13 under different (**a**) TAN concentrations (mg-N/L) and (**b**) pH.

The nitrifying activity of HG-0.55, which has a relatively thin thickness, was higher than that of HG-1.13. The nitrifying activity was extremely inhibited in the TAN concentration of 200 mg-N/L compared to that of 100 mg-N/L, and it weakly decreased as the TAN concentration increased in the range of 200–500 mg-N/L (Fig. 1a). By the calculation of Eq. (5), as the TAN concentration increases from 50 to 500 mg-N/L, the FA concentration increases from 0.204 to a maximum of 2.036 mg-FA/L. The FA in wastewater could significantly reduce the nitrifying activity of major microorganisms responsible for nitrogen treatment. This occurs through the degradation of EPS and cell envelopes by disrupting their chemical bonds and electrostatic interactions^30,52^. The high nitrifying activity was observed at pH 8 and decrease in the other ranges (Fig. 1b). When the pH is raised from 8 to 9, the nitrifying activity is significantly suppressed due to a sharp increase in FA concentration from 3.928 to 29.017 mg-FA/L. In contrast, under acidic conditions, free nitrous acid (HNO_3_) is formed which inhibits the activity of nitrifying bacteria^53^. The optimum pH range for nitrifying bacteria is from 7.2 to 8.2. Deviations from this range can impair microbial functions or lead to alteration of enzyme reaction mechanisms and eventual demise of the bacteria^30,54^.

Through preliminary feasibility tests, it was demonstrated that TAN concentration and pH have a significant impact on specific nitrifying activity. Based on these results, NH_4_^+^-N concentration of 100 mg-N/L and pH of 8 were determined as the optimal conditions for maximizing specific nitrifying activity. These conditions were used as the center point in the RSA in section “Model development of ammonia-oxidizing activity”, and the combined impact of TAN concentration and pH on nitrification activity was analyzed.

### Model development of ammonia-oxidizing activity

The RSA was carried out using a multiple regression approach with TAN concentration and pH as the independent variables, as shown in Table 2. The SOUR was measured to perform this modeling, aiming to analyze the interactive effects of TAN concentration and pH on nitrification activity. All observed responses were simultaneously fitted to the first-order, second-order, and partial cubic models. The first-order model is a linear model with cross terms and does not account for surface curvature, and it is commonly used in the initial stage of experiments^55,56^. Seven trials including center points (trials 1–5 in Table 2) were conducted to test the adequacy of the first-order model in describing the response surface of nitrate-reducing activity (Eqs. 7 and 8).

7 $${\text{For}}\;{\text{HG - }}0.{55}: \, \eta_{{{\text{HG}} - 0.{55},{\text{ first}} - {\text{order model}}}} = { 5}.{4186} \times {1}0^{{ - {3}}} + { 5}.{385}0 \times {1}0^{{ - {6}}}_{ } x_{1} + { 4}.{32}00 \times {1}0^{{ - {4}}}_{ } x_{2}$$

8 $${\text{For}}\;{\text{HG - 1}}.{13}: \, \eta_{{{\text{HG}} - {1}.{13},{\text{ first}} - {\text{order model}}}} = { 2}.{4586} \times {1}0^{{ - {3}}} + { 9}.{25}00 \times {1}0^{{ - {7}}} x_{1} + { 4}.{6875} \times {1}0^{{ - {4}}} x_{2}$$

**Table 2:** Experimental design for composite design (CCD) and the corresponding results of SOUR experiment.

Trials	Conditions of variables		SOUR (mg-DO/L mL-PVA min)	
	TAN concentration (mg/L) (code)	pH (code)	HG-0.55	HG-1.13
1	150 (+ 1)	9 (+ 1)	0.00478 (^a^Est: 0.00499)	0.00254 (0.00242)
2	150 (+ 1)	7 (–1)	0.00453 (0.00474)	0.00202 (0.00190)
3	50 (– 1)	9 (+ 1)	0.00486 (0.00507)	0.00286 (0.00275)
4	50 (– 1)	7 (–1)	0.00338 (0.00359)	0.00151 (0.00139)
^b^5	100 (0)	8 (0)	0.00723 ± 0.00080 (0.00723)	0.00278 ± 0.00027 (0.00276)
6	170.7 (+ 2^½^)	8 (0)	0.00789 (0.00768)	0.00294 (0.00306)
7	29.3 (– 2^½^)	8 (0)	0.00584 (0.00563)	0.00201 (0.00213)
8	100 (0)	9.4 (+ 2½)	0.00233 (0.00211)	0.00119 (0.00132)
9	100 (0)	6.6 (– 2^½^)	0.00338 (0.00316)	0.00187 (0.00200)

^a^Est: Estimated SOUR value by the regression model developed in this study.

^b^The experiment was repeated five times, and the response represents average values.

Based on the results of the first-order response surface analysis, the first-order model of HG-0.55 exhibits a low R-squared value of 0.089 and a non-significant P-value of 0.8291, indicating that the model is not statistically valid (Eq. 7). Similarly, the first-order model of HG-1.13 has an R-squared value of 0.499 and a non-significant P-value of 0.2513, suggesting that the model's adequacy is poor (Eq. 8). An inadequate first-order model suggests that there is curvature in the response surface within the explored region, which can be addressed by using higher-order models to locate the turning point. The second-order model and partial cubic models were fitted with 13 trials, including six augmented points (trials 6–9 in Table 2). The partial cubic model was examined by adding the terms of x_1_^2^x_2_ and x_1_x_2_^2^. In order to provide a comprehensive analysis, the R-squared and P-values were calculated for the second-order model as well. For HG-0.55, the R-squared value was found to be 0.894 with a corresponding P-value of 0.0026. Similarly, for HG-1.13, the R-squared value was determined to be 0.634 with a corresponding P-value of 0.1401. Based on the comparison of first-order, second-order, and partial cubic models, the most suitable model was determined to be the partial cubic model, represented by Eqs. (9) and (10).

9 $$\begin{aligned} {\text{For}}\;{\text{HG - }}0.{55}: \, \eta_{{{\text{HG}} - 0.{55},{\text{ partial cubic model}}}} & = { 7}.{2264} \times {1}0^{{ - {3}}} + { 1}.{4491} \times {1}0^{{ - {5}}} x_{1} \\ & \;\;{-}{ 3}.{7393} \times {1}0^{{ - {4}}} x_{2} {-}{ 6}.{1}0{5}0 \times {1}0^{{ - {6}}} x_{1} x_{2} \\ & \;\;{-}{ 1}.{1478} \times {1}0^{{ - {7}}} x_{1}^{2} {-}{ 2}.{3413} \times {1}0^{{ - {3}}} x_{2}^{2} \\ & \;\; {-}{ 9}.{1}0{58} \times {1}0^{{ - {6}}} x_{1} x_{2}^{2} + { 3}.{2267} \times {1}0^{{ - {7}}} x_{1}^{2} x_{2} \\ \end{aligned}$$

10 $$\begin{aligned} {\text{For}}\;{\text{HG - 1}}.{13}: \, \eta_{{{\text{HG}} - {1}.{13},{\text{ partial cubic model}}}} & = { 2}.{56}0{2} \times {1}0^{{ - {3}}} + { 6}.{57}00 \times {1}0^{{ - {6}}} x_{1} \\ & \;\;{-}{ 2}.{4286} \times {1}0^{{ - {4}}} x_{2} {-}{ 4}.{175}0 \times {1}0^{{ - {6}}} x_{1} x_{2} \\ & \;\;{-}{ 3}.{2153} \times {1}0^{{ - {8}}} x_{1}^{2} {-}{ 5}.{6165} \times {1}0^{{ - {4}}} x_{2}^{2} \\ & \;\;{-}{ 5}.{645}0 \times {1}0^{{ - {6}}} x_{1} x_{2}^{2} + { 2}.{8464} \times {1}0^{{ - {7}}} x_{1}^{2} x_{2} \\ \end{aligned}$$

The partial cubic model provided the most suitable formulas for HG-0.55 and -1.13. The R-squared values for HG-0.55 and -1.13 were 0.933 and 0.907, respectively, indicating a good fit. The corresponding p-values for HG-0.55 and -1.13 were 0.011 and 0.024, respectively, which were statistically significant. The lack of fit for both models was not found significant at the 5% level.

**Figure 2:**
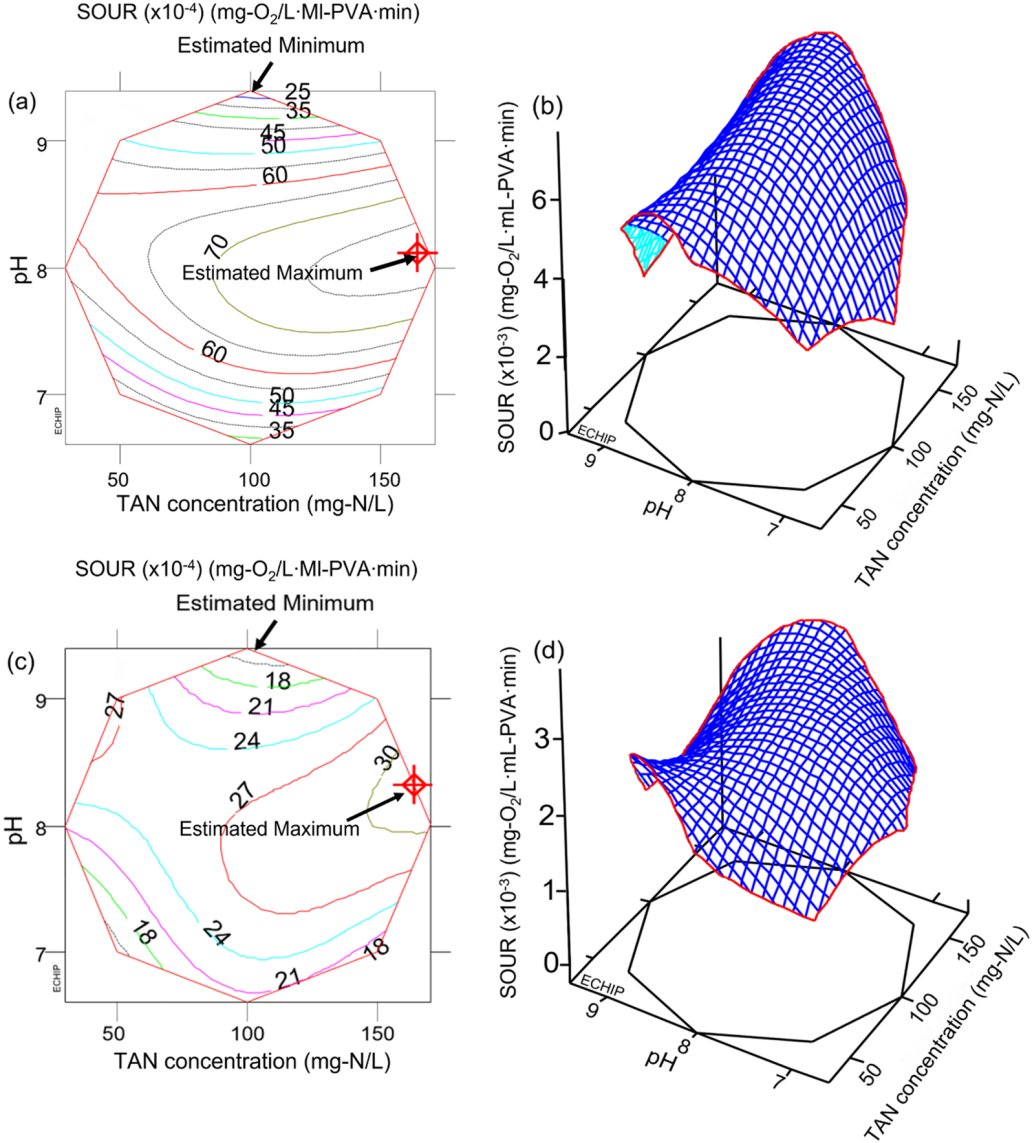
Two- and three-dimensional contour plots of the partial cubic model: (**a**) and (**b**) for HG-0.55, (**c**) and (**d**) for the HG-1.13.

For HG-0.55, the optimal conditions were found to be 167.54 mg-N/L of TAN and pH 8, as determined by Eq. (7), which were well within the explored boundary (Fig. 2a and b). Meanwhile, for HG-1.13, the optimal conditions were determined to be 165.52 mg-N/L of TAN and pH 8.25, as identified by Eq. (8), which were located near the edge of the explored region (Fig. 2c and d). The predicted model outputs under these conditions were 0.00768 and 0.00317 mg-O_2_/L mL-PVA min for HG-0.55 and -1.13, respectively. In general, the thin HG-0.55 exhibited a SOUR value that is about 2–3 times higher than that of HG-1.13. The reaction contours of nitrifying activity exhibit significant changes along the pH axis compared to the formation of a long elliptical surface along the TAN concentration axis. This indicates that the sensitivity of the model to pH is much higher than to TAN concentration. This observation is consistent with the fact that pH has a more important impact on the activity of nitrifying bacteria than TAN concentration^57^. A detailed comparison of HG-0.55 and -1.13 is discussed in section “Impact of TAN concentration and pH on ammonia-oxidizing activity: a simulation study” through a simulation study.

### Impact of TAN concentration and pH on ammonia-oxidizing activity: a simulation study

**Figure 3:**
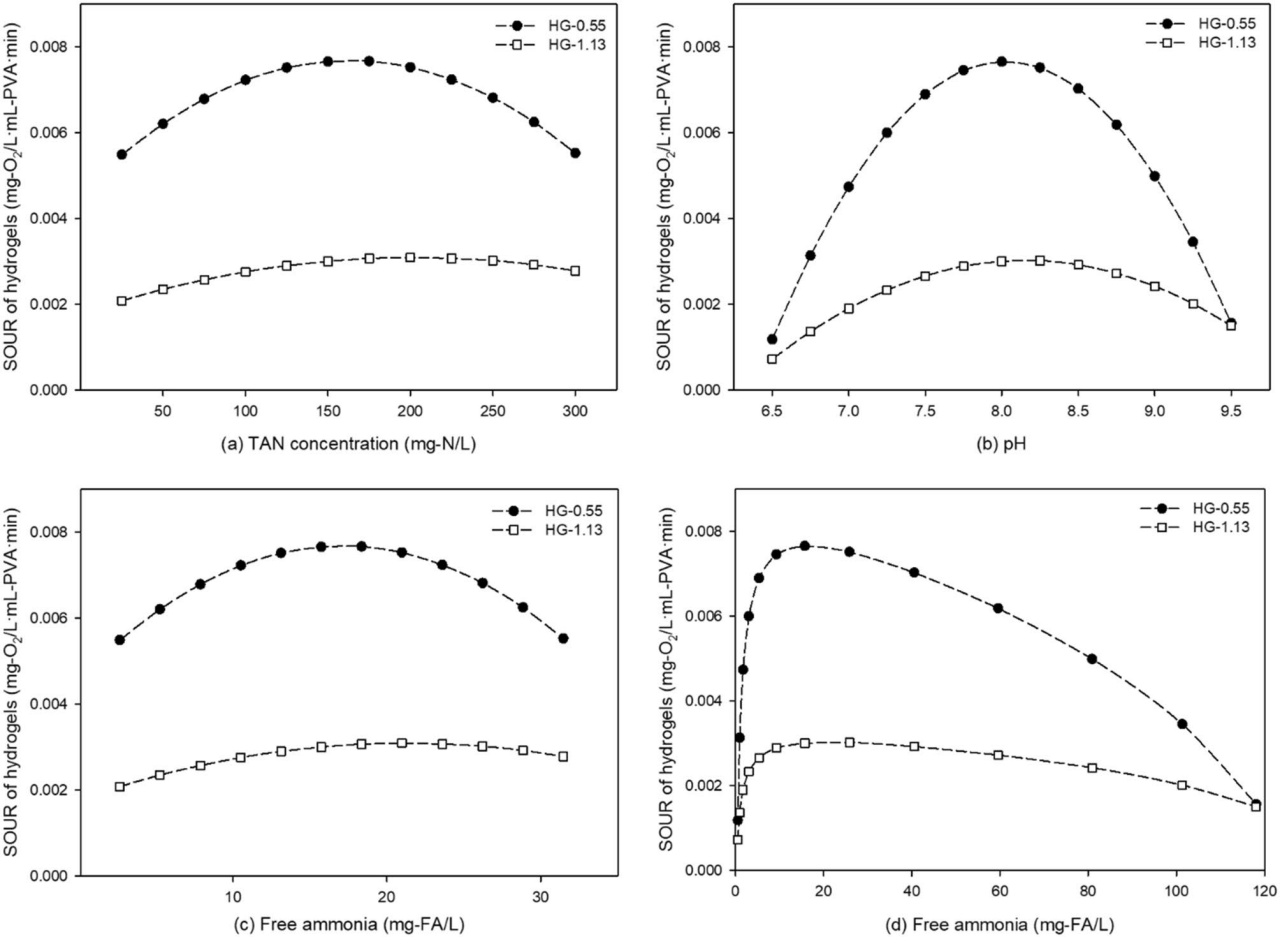
Simulation of SOUR values using the partial cubic model for HG-0.55 and -1.13 with varying thickness: (**a**) TAN concentration (mg-N/L), (**b**) pH, (**c**) free Ammonia (based on TAN concentration), and (**d**) free ammonia (based on pH).

The nitrifying activity of HG-0.55 and HG-1.13 was examined in relation to TAN concentration and pH, utilizing regression Eqs. (9) and (10), respectively, from partial cubic models for individual responses to these variables (Fig. 3). For the simulation of TAN concentration, the pH was held constant at 8, while TAN concentrations were evaluated at intervals of 25 mg-N/L, ranging from 25 to 300 mg-N/L (Fig. 3a). Conversely, when simulating the effects of pH, the TAN concentration was fixed at 150 mg-N/L, and the pH was evaluated at intervals of 0.25, spanning from 6.5 to 9.5 (Fig. 3b). The nitrifying activity of HG-0.55 and HG-1.13 was then determined by the resulting SOUR values, which were obtained by inputting the independently set variables (TAN concentration and pH) into the regression equations as independent variables.

**Figure 4:**
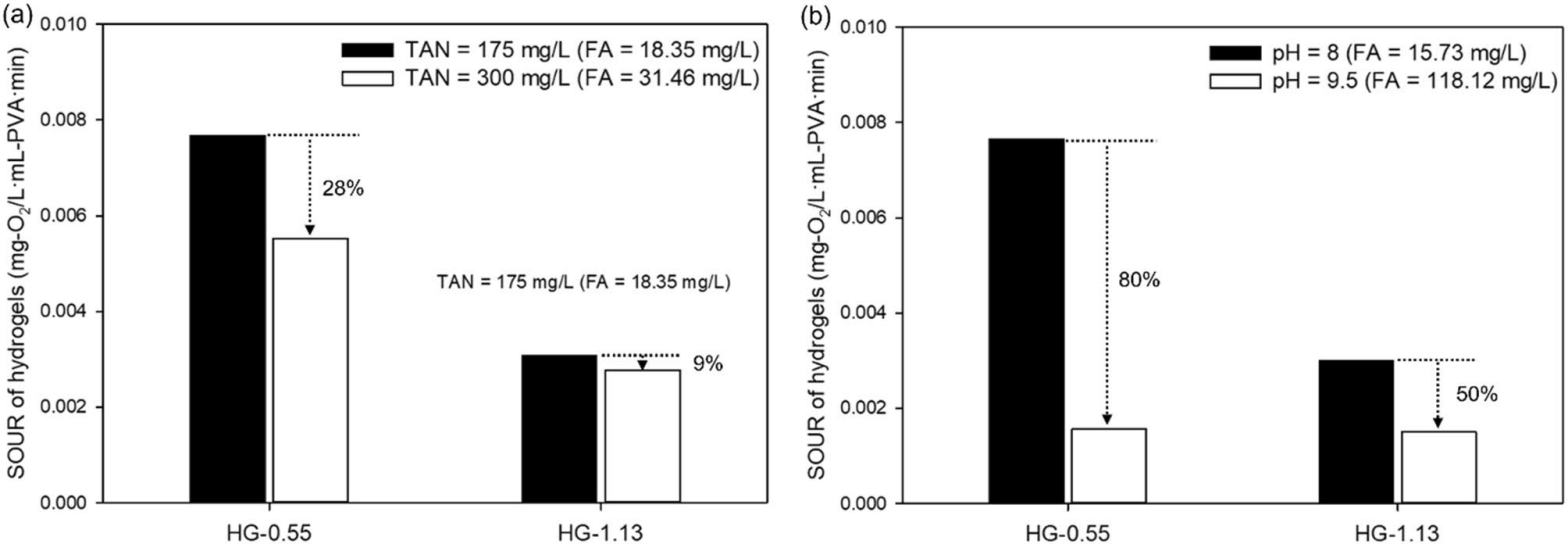
Estimating the effect of optimal and maximum (**a**) TAN concentration (at pH 8) and (**b**) pH (at TAN concentration: 150 mg/L) values on SOUR for HG-0.55 and HG-1.13.

The optimal TAN concentration for HG-0.55 and HG-1.13 was found to be 175 mg-N/L. At this concentration, HG-0.55 exhibited a SOUR value of 0.00767 mg-O_2_/L mL-PVA min, while HG-1.13 had a SOUR value of 0.00307 mg-O_2_/L mL-PVA min (Fig. 3a). The optimal SOUR value discussed in section “Model development of ammonia-oxidizing activity” refers to the value achieved under optimal conditions of both TAN concentration and pH, as illustrated by the contour plots of the partial cubic model. In contrast, the SOUR value mentioned in this section corresponds to the value obtained at the TAN concentration that produces the highest SOUR value when the pH is held constant at 8. The simulation results obtained through RSA demonstrate a clear inverse correlation between SOUR and susceptibility to FA toxicity. Compared to the TAN concentration of 150 mg-N/L, increasing the TAN concentration to 300 mg-N/L resulted in a 100% increase in FA concentration from 15.73 to 31.46 mg-FA/L, while the SOUR values of HG-0.55 and -1.13 decreased by 28% and 9% (Fig. 4a), respectively. The optimal pH for both HG-0.55 and HG-1.13 was found to be 8 at a TAN concentration of 150 mg/L, with SOUR values of 0.00766 mg-O_2_/L mL-PVA min and 0.003 mg-O_2_/L mL-PVA min, respectively (Fig. 3b). The high pH resulted in drastic inhibition of nitrifying activity compared to TAN inhibition. At pH 9.5, the concentration of FA increased to 118.12 mg-FA/L, resulting in a reduction of 80% for the SOUR value in the case of HG-0.55 and 50% in the case of HG-1.13 (Fig. 4b).

Overall, HG-1.13 exhibited less sensitivity to FA inhibition caused by pH and TAN concentration increases compared to HG-0.55. Nitrification is highly responsive to pH, with optimal conditions reported to exist within the narrow range of 7 to 8. Previous studies have observed that the activity of AOB completely ceases at pH levels below 6^58,59^. On the other hand, high FA can impede enzyme activity and result in reduce acid production^20^. It has been reported that FA concentrations higher than 50–100 mg-FA/L negatively impact the activity of AOB. Meanwhile NOB is inhibited at FA concentrations, ranging from 0.1 to 10 mg-FA/L^60^. In this study, the FA concentration of 15.7 mg-FA/L generated under the optimal active conditions of TAN concentration of 150 mg-N/L and pH 8 is considered sufficient for the survival of AOB, but NOB may still be inhibited (Fig. 3c and d). In contrast, under pH 9 conditions, the calculated FA concentration was 80.9 mg-FA/L, creating an environment in which the activities of both AOB and NOB were inhibited.

The surface area of HG-0.55 per one gel piece is 5.875 cm^2^, while that of HG-1.13 is 8.75 cm^2^. When the same volume of HGs is applied, HG-0.55 has 1.34 times higher specific area (surface area/volume) than HG-1.13. The depth of substrate penetration could vary depending on factors such as particle size and shape, pore size, and biofilm thickness of microorganisms. Previous studies reported that the maximum depth of substrate penetration in granular biomass and cryogel is limited to 1.3 mm^61,62^. Areas beyond 1.3 mm deep are regarded as dead space, and consequently, decreasing the gel thickness results in a larger active area for carriers of equal volume. The simulation results of the RSA indicate that when the thickness of hydrogel is relatively thin, the nitrification activity is higher under optimal conditions; however, it is more sensitive to changes in FA concentration. If the thickness of hydrogel is thick, it is suspected that the influence on nitrifying bacteria would decrease as FA cannot penetrate deep into the hydrogel. Considering the limitation of existing studies on this subject, further research is essential to better understand the interplay between hydrogel thickness, ammonia-oxidizing activity, and FA.

### Nitrification in continuous mode

**Figure 5:**
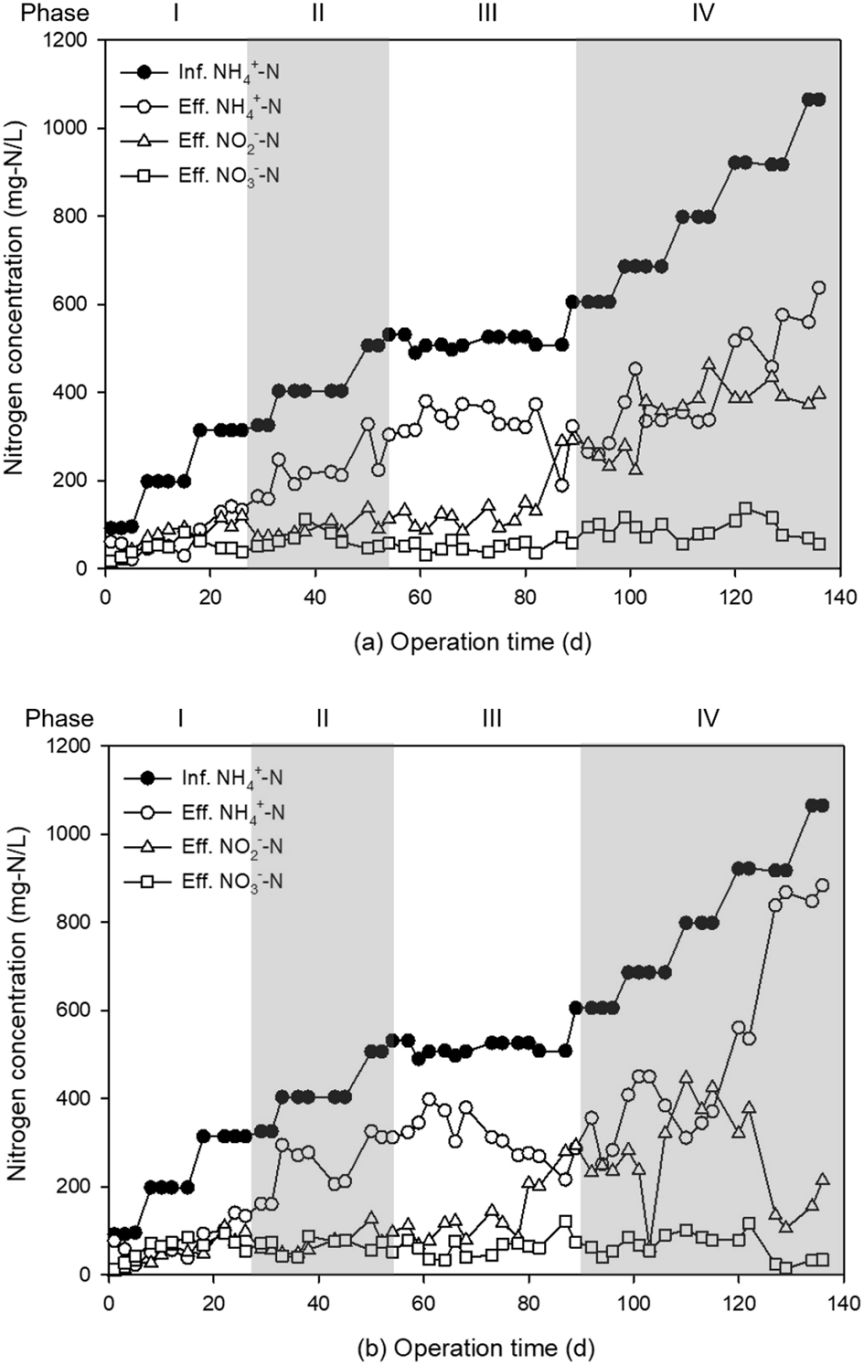
Nitrogen concentration of influent and effluent in nitrifying bioreactors for evaluating the PN performance of reactors with (**a**) HG-0.55 and (**b**) -1.13.

Two lab-scale bioreactors were operated to assess the PN applicability over a 136-day period, as illustrated in Fig. 5. In the continuous mode, influent containing synthetic wastewater with low to high concentrations of TAN was gradually fed. In the continuous process for treating wastewater, a stepwise TAN increase strategy that prevents the accumulation of FA has a significant impact on stabilizing the process. In Phase I, the initial TAN of 100 mg-N/L in both bioreactors was oxidized by 80% within 5 days. Additionally, 300 mg-N/L of NH_4_^+^–N was oxidized by over 50% within 26 days, which attests to the successful achievement of nitrifying efficiency. The NO_3_^−^–N concentrations of effluent of reactors with HG-0.55 and -1.13 were measured to be 37.7 mg-N/L and 53.7 mg-N/L, respectively. This result indicates that the activity of NOB was more inhibited in HG-0.55 compared to HG-1.13. During Phase II, two operational conditions were concurrently altered: TAN concentration was increased from 300 to 500 mg-N/L, while the HCO_3_^−^ to NH_4_^+^-N alkalinity ratio was decreased to a 1:1 molar ratio. As a result of these changes, the activity of AOB was decreased by approximately 50% in both reactors. In Phase III, the operational temperature was adjusted to 30–35 °C to further inhibit the NOB activity. After the temperature increase, the ARR of reactor with HG-0.55 decreased by approximately 34% from 0.226 ± 0.006 kg-N/m^3^ day to 0.150 ± 0.021 kg-N/m^3^ day at an influent TAN concentration of 500 mg-N/L between 59 and 68 days. The NRR also decreased by 51% from 0.102 ± 0.020 kg-N/m^3^ day to 0.050 ± 0.017 kg-N/m^3^ day during the same period. The greater decrease in NRR compared to ARR suggests that NOB was more inhibited than AOB due to the temperature increase. The activity of nitrifying bacteria in HG-1.13 was slightly more inhibited than that in HG-0.55, as evidenced by a decrease in ARR and NRR of 36% and 57%, respectively. After 68 days, to recover the activity of AOB, the alkalinity was increased to 1.2 times the HCO_3_^−^ to NH_4_^+^–N. As a result, the ARR of HG-0.55 and HG-1.13 improved to 0.317 ± 0.030 kg-N/m^3^ day and 0.284 ± 0.013 kg-N/m^3^ day, respectively, after 87 days of operation.

In Phase IV, the ability of HG-0.55 and -1.13, with a 10% packing ratio, to endure high-strength FA was tested by gradually increasing the influent TAN concentration to 1000 mg-N/L. When the TAN concentration of influent was 600 mg-N/L and the ALR was 0.60 kg-N/m^3^ day, the ARR of reactor with HG-0.55 was 0.33 kg-N/m^3^ day, while that of HG-1.13 was 0.30 kg-N/m^3^ day (Fig. S3). Both reactors maintained a stable ARR while oxidizing over 50% of NH_4_^+^-N as the TAN concentration increased to a maximum of 800 mg-N/L. In the TAN concentration range of 600–800 mg-N/L, the NH_4_^+^–N to NO_2_^−^–N ratio of reactor with HG-0.55 was 0.97, which was more suitable for PN than HG-1.13, which had a ratio of 0.79. At a TAN concentration of 900 mg/L, the effluent concentrations of NH_4_^+^–N, NO_2_^−^–N, and NO_3_^−^–N were 457.6, 433.9, and 115.4 mg-N/L, respectively, in the case of the reactor with HG-0.55 (Fig. 5a). The ARR was 0.40 ± 0.05 kg-N/m^3^ day under these conditions (Fig. S3). This reactor is maintained a ratio of NH_4_^+^–N to NO_2_^−^–N at 0.95. However, that exhibited decreased ammonia-oxidation efficiency of 43.77 ± 5.21% when the TAN concentration was increased to 1000 mg-N/L. In contrast, the reactor utilizing HG-1.13 exhibited a noteworthy decrease in ammonia-oxidation efficiency at 7.02 ± 2.38% when the TAN concentration surpassed 900 mg-N/L (Fig. 5b). During the operation period of 115–129 days, when the ALR increased from 0.78 to 0.95 kg-N/m^3^ day, the FA concentration of effluent in reactor with HG-0.55 increased from 14.77 to 103.54 mg-FA/L, resulting in a FA accumulation rate of 0.0179 kg-FA/m^3^ day (Fig. S4a). In contrast, effluent in reactor with HG-1.13 had a much faster FA accumulation rate of 0.0516 kg-FA/m^3^ day, with FA concentration increasing from 17.33 to 204.01 mg-FA/L (Fig. S4b). The SOUR obtained from batch mode can serve as an effective semi-quantitative indicator for predicting the PN performance in continuous mode. However, FA susceptibility does not have a significant impact in continuous mode. Although HG-0.55 was more sensitive to FA toxicity than HG-1.13, its high ammonia-oxidation efficiency in continuous mode tests prevented the accumulation of FA concentration inside the reactor. Therefore, despite being more sensitive to FA toxicity, HG-0.55 inoculated reactor with a high surface area and high ammonia oxidation efficiency was determined to be suitable for treating high-strength wastewater containing high ammonia.

## Conclusions

This study quantitatively evaluated the differences in nitrifying activity of entrapped nitrifying bacteria on carriers of different thicknesses. The nitrifying activity was evaluated by analyzing SOUR at various ranges of TAN concentration and pH, and RSA was conducted using SOUR values as response variables. The regression equation derived from the partial cubic model was successful, indicating that it could be well utilized in the nitrification process. HG-0.55 and HG-1.13 had the highest nitrification activity at around 165 mg-N/L of TAN concentration and pH 8, with respective activity values of 0.00768 and 0.00317 mg-O_2_/L mL-PVA min. The simulation results based on RSA values indicated that HG-0.55 had 254% higher nitrification activity than HG-1.13. However, when the pH decreased from the optimal pH to 6.5, the nitrification activity of HG-1.13 decreased by 76%, while that of HG-0.55 decreased by 85%, suggesting that HG-1.13 was less sensitive to pH-related toxicity. In the continuous mode test, reactor with HG-0.55 had overall higher ammonia-oxidation efficiency and a more suitable NH_4_^+^–N to NO_2_^−^–N ratio for PN than that of HG-1.13. HG-0.55 maintained stable PN performance at a TAN concentration of 900 mg-N/L, with an ARR of 0.40 ± 0.05 kg-N/m^3^ day and an NH_4_^+^–N to NO_2_^−^–N ratio of 0.95. However, reactor using HG-1.13 exhibited decreased ammonia-oxidation efficiency when the influent TAN concentration surpassed 900 mg-N/L. Due to the high ammonia-oxidation efficiency of reactor with HG-0.55, the accumulation of FA was retarded, resulting in higher PN performance in high-concentration wastewater compared to that of HG-1.13.

## Supplementary Information

Supplementary Figures.

## Data Availability

The data sets analyzed during the current study are available from the corresponding author on reasonable request.
