# Author Correction: Thickness of hydrogel for nitrifying biomass entrapment determines the free ammonia susceptibility differently in batch and continuous modes

**DOI:** 10.1038/s41598-025-95240-2

**Published:** 2025-04-10

**Authors:** Minsu Song, Meng Yuan, Sanghyun Jeong, Hyokwan Bae

**Affiliations:** 1 Department of Civil and Environmental Engineering, Pusan National University, Busan, 46241 Republic of Korea; 2 Department of Urban and Environmental Engineering, Ulsan National Institute of Science and Technology (UNIST), 50 UNIST‑gil, Eonyang‑eup, Ulju‑gun, Ulsan, 44919 Republic of Korea; 3 Graduate School of Carbon Neutrality, Ulsan National Institute of Science and Technology (UNIST), 50 UNIST‑gil, Eonyang‑eup, Ulju‑gun, Ulsan, 44919 Republic of Korea

Correction to: *Scientific Reports* 10.1038/s41598-023-36507-4, published online 08 June 2023

The original version of this Article contained an error in Reference 4, which was incorrectly given as:

Vickers, N. J. Animal communication: When i’m calling you, will you answer too? Curr. Biol. 27, R713–R715. 10.1016/j.envres.2022.112944 (2017).

The correct reference is listed below:

Masindi, V. et al. Emerging remediation potentiality of struvite developed from municipal wastewater for the treatment of acid mine drainage. *Environ. Res.* **210**, 112944. 10.1016/j.envres.2022.112944 (2022).

The original Article has been corrected.

