# Supplementary material for: Thickness of hydrogel for nitrifying biomass entrapment determines the free ammonia susceptibility differently in batch and continuous modes
Source: Sci Rep. 2023 Jun 8;13:9353. doi: 10.1038/s41598-023-36507-4 (PMC10250323; doi:10.1038/s41598-023-36507-4)
Supplement: Supplementary file 1 — Supplementary Figures. [file 41598_2023_36507_MOESM1_ESM.docx]

**Thickness of hydrogel for nitrifying biomass entrapment determines the free ammonia susceptibility differently in batch and continuous modes**

Minsu Song^a, 1^, Meng Yuan^a, 1^, [Sanghyun Jeong](https://www.sciencedirect.com/author/57200597289/sanghyun-jeong)^a^, Hyokwan Bae^b, c,^ *

^a^Department of Civil and Environmental Engineering, Pusan National University, 63 Busandeahak-ro, Geumjeong-Gu, Busan 46241, Republic of Korea

^b^Department of Urban and Environmental Engineering, Ulsan National Institute of Science and Technology (UNIST), 50 UNIST-gil, Eonyang-eup, Ulju-gun, Ulsan 44919, Republic of Korea

^c^Graduate School of Carbon Neutrality, Ulsan National Institute of Science and Technology (UNIST), 50 UNIST-gil, Eonyang-eup, Ulju-gun, Ulsan 44919, Republic of Korea

*Corresponding author: Hyokwan Bae, Ph.D.

Department of Urban and Environmental Engineering, Ulsan National Institute of Science and Technology (UNIST), 50 UNIST-gil, Eonyang-eup, Ulju-gun, Ulsan 44919, Republic of Korea

E-mail: [hyokwan.bae@unist.ac.kr](mailto:hyokwan.bae@unist.ac.kr)

Tel: +82-52-217-2801

Fax: +82-52-217-2859

ORCID: 0000-0002-2422-9411

^1^These authors contributed equally to this work


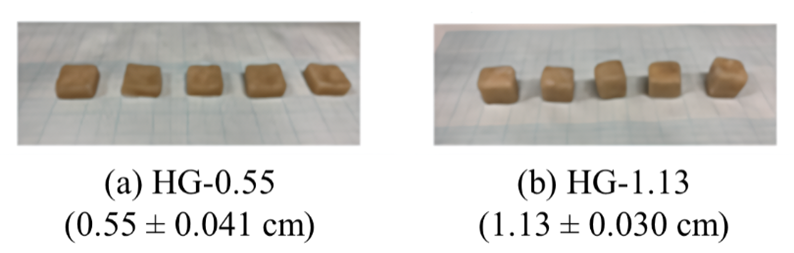


**Fig. S1.** The average thicknesses of (a) HG-0.55 and (b) -1.13 entrapping nitrifying bacteria.


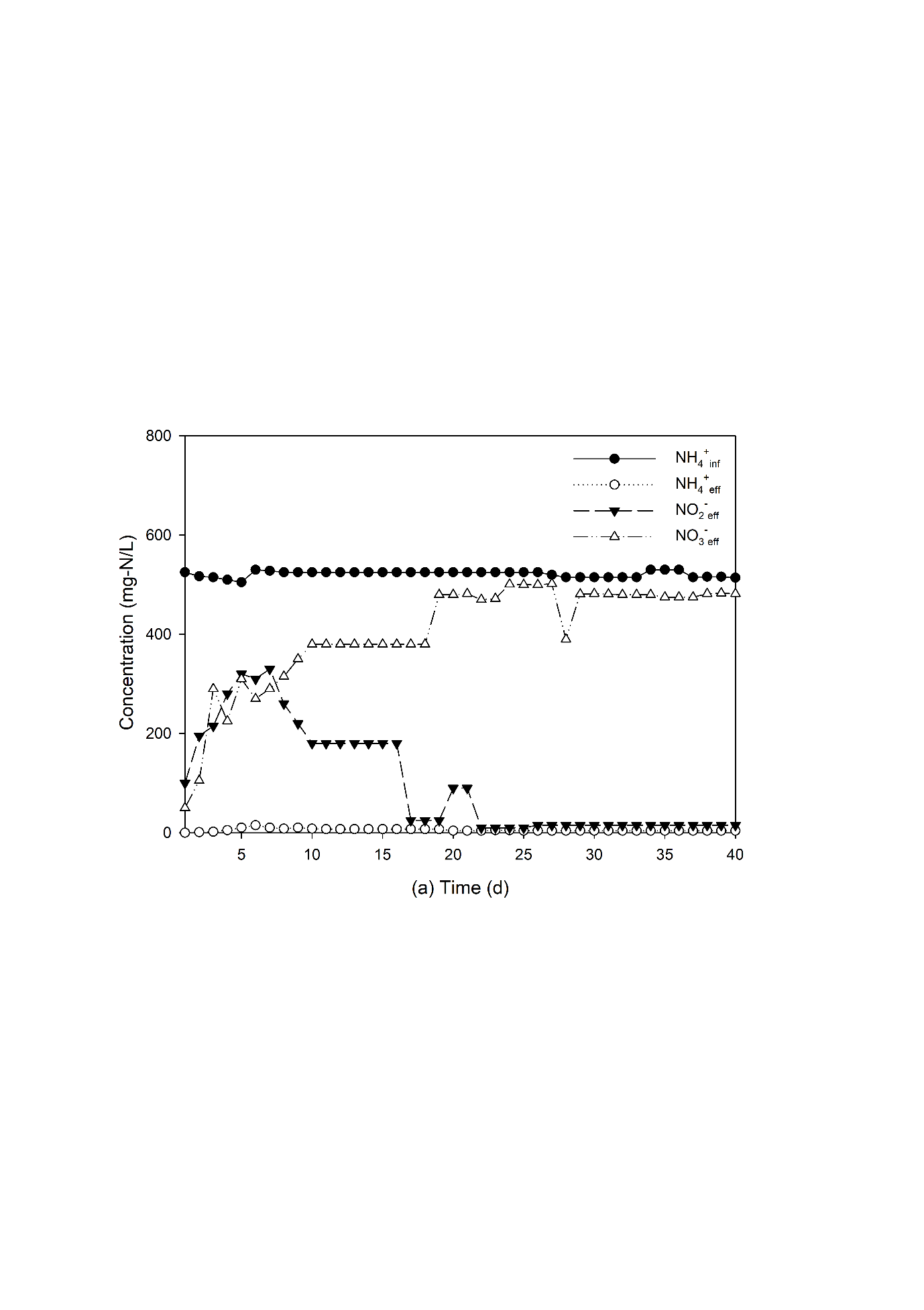

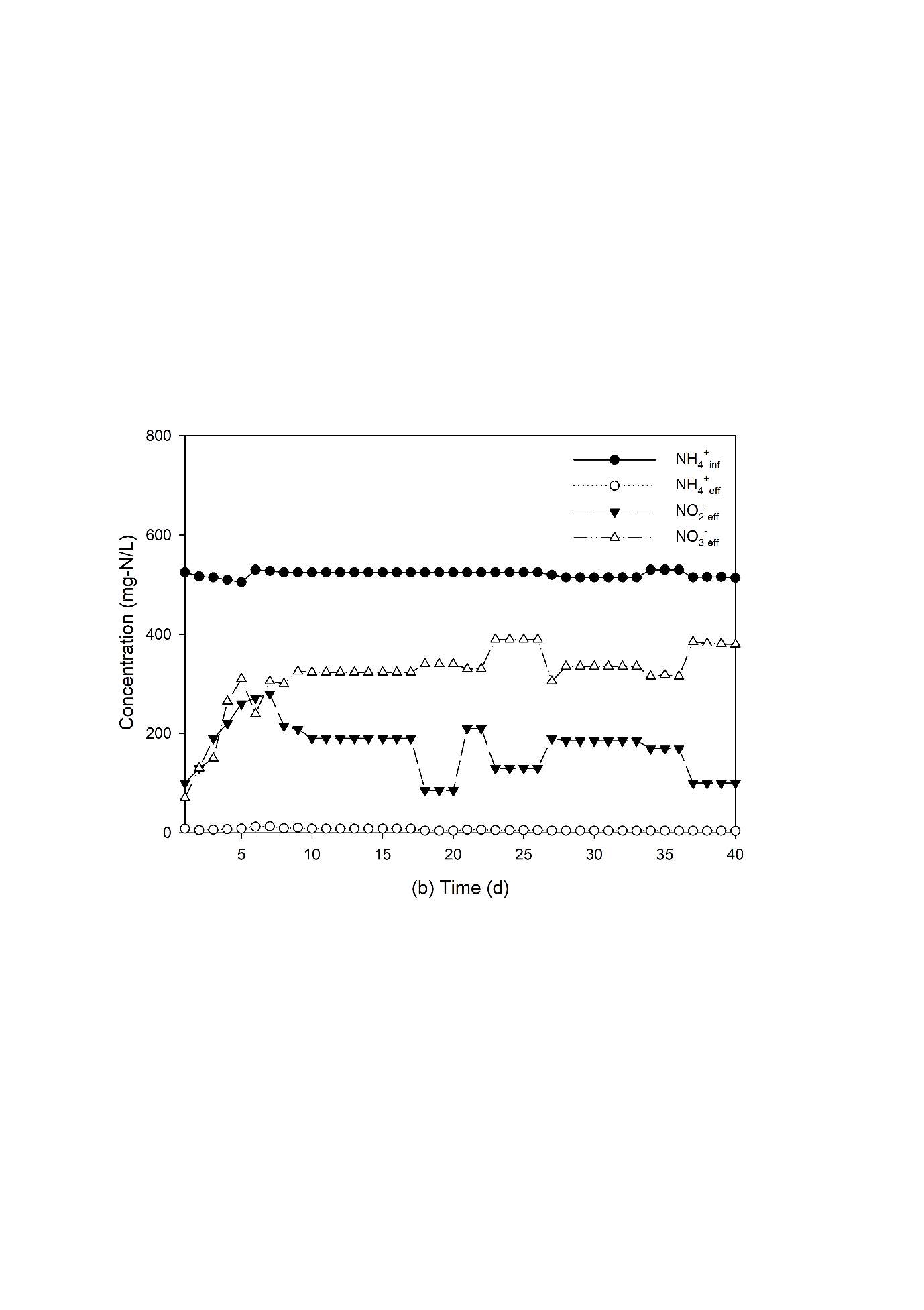


**Fig. S2.** Nitrifying efficiency of (a) HG-0.55 and (b) -1.13 during the enrichment period.


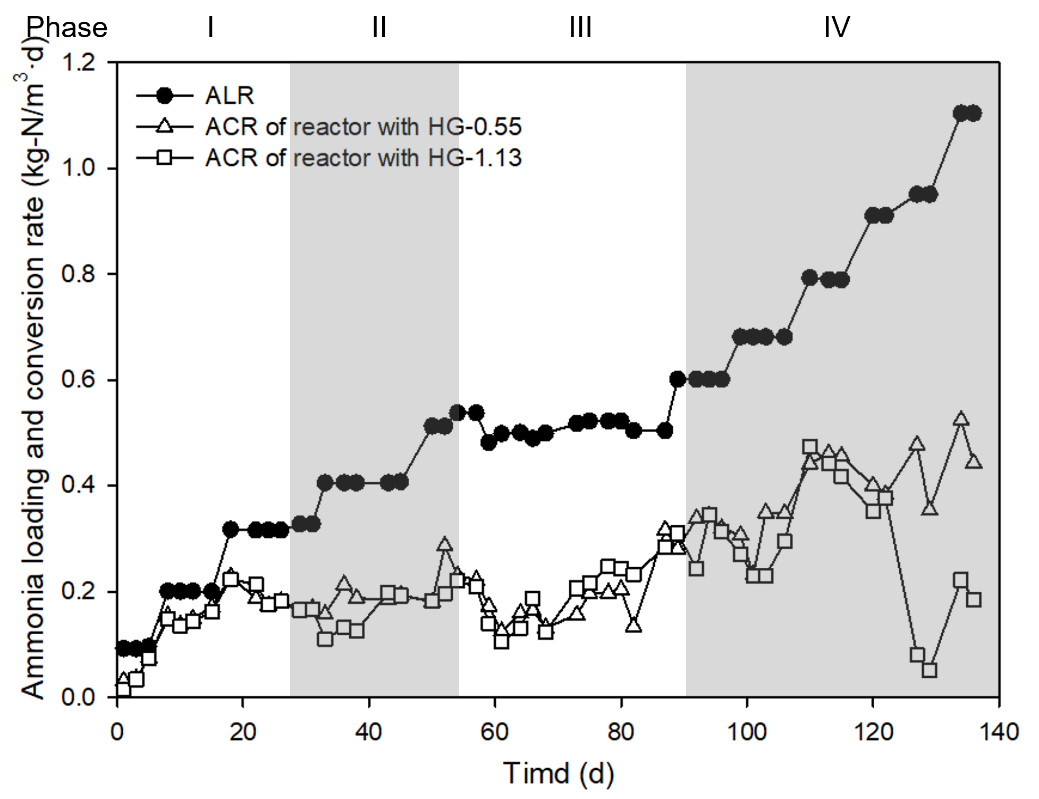


**Fig. S3.** Ammonia and nitrite conversion rate of nitrifying bioreactors for evaluating the PN performance of reactors with HG-0.55 and -1.13.


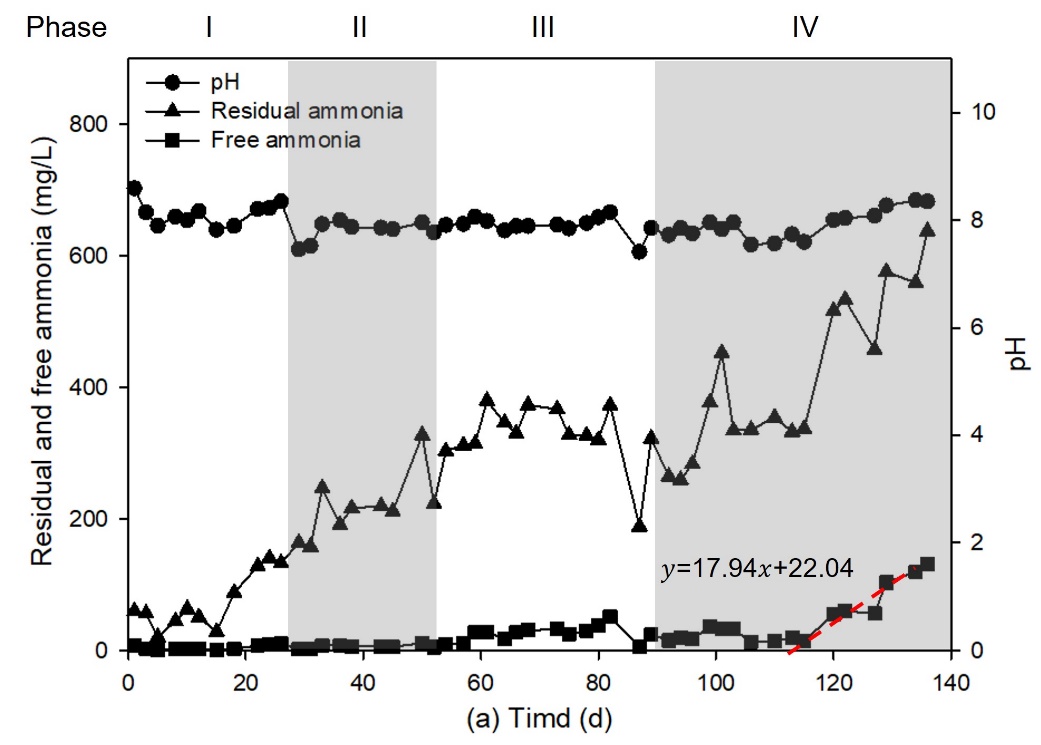

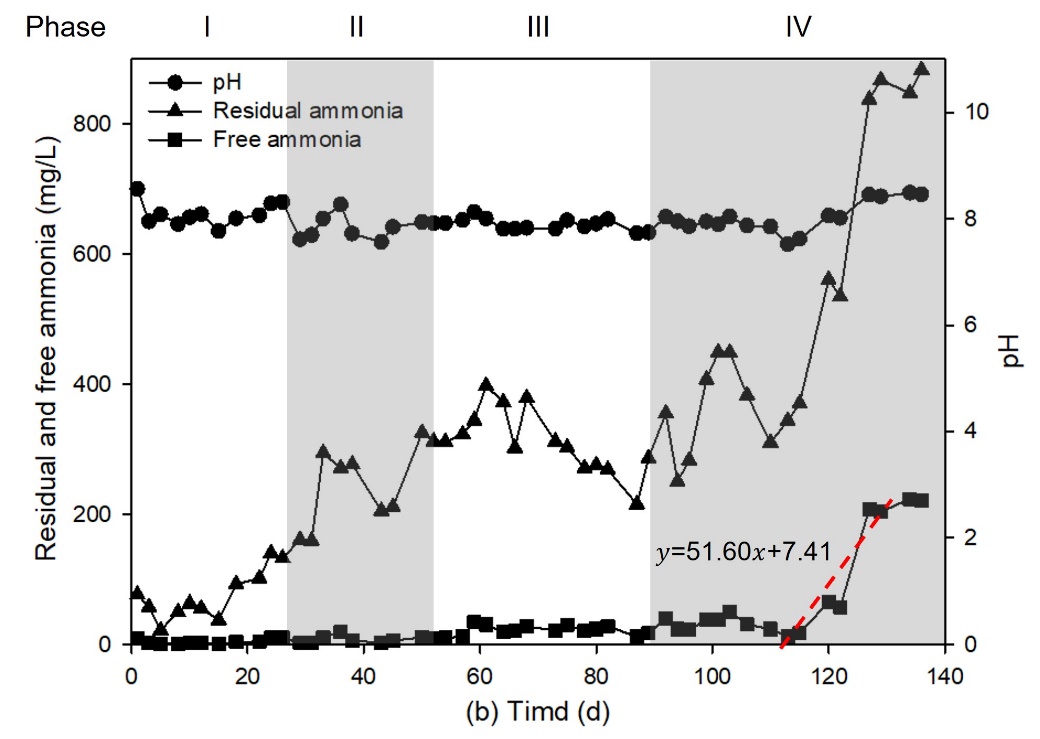


**Fig. S4.** pH, residual, and free ammonia concentration of nitrifying bioreactors for evaluating the PN performance of reactors with (a) HG-0.55 and (b) -1.13 (including regression equations for calculating the rate of FA increase during the 115–129 days period).
